# Beneficial effects of saw palmetto (*Serenoa repens*) fruit extract on the urinary symptoms of healthy Japanese adults with possible lower urinary tract symptoms: A randomized, double-blind, placebo-controlled study

**DOI:** 10.1177/02601060241265389

**Published:** 2024-07-23

**Authors:** Mai Kimura, Ikuya Ishii, Asami Baba, Tsuyoshi Takara

**Affiliations:** 1 YAWATA CORPORATION, Yonago, Tottori, Japan; 2 ORTHOMEDICO Inc., Bunkyo-ku, Tokyo, Japan; 3 Medical Corporation Seishinkai, Takara Clinic, Shinagawa-ku, Tokyo, Japan

**Keywords:** Saw palmetto fruit extract, urinary symptoms, quality of life, urinary frequency, urine leakage

## Abstract

Saw palmetto extract (SPE) is the most commonly used supplement for the treatment of lower urinary tract symptoms (LUTS), but most evidence is for those with LUTS, and little data is verifying its effectiveness for those who do not have the disease but are troubled by symptoms. The purpose of this study was to examine the effect of SPE on the improvement of urinary frequency problems that present stress due to urinary urgency in daily life, among healthy Japanese adults aged ≥50 years who are not diagnosed with benign prostatic hyperplasia or overactive bladder. They were randomly assigned to the SPE group or placebo group (34 participants per group) using a computerized random number generator. Each participant was instructed to take one capsule containing SPE (320 mg) or placebo every day for 12 weeks. Subjective symptoms were assessed using the overactive bladder questionnaire, and the score of symptom bother by frequent urination during the daytime hours was set as the primary outcome. The other outcomes were subjective urinary symptoms and urinary frequencies. The final efficacy analysis dataset was per protocol set, and 33 participants in each group were analyzed. After SPE intervention for 12 weeks, the score of symptom bother by frequent urination during the daytime hours was significantly improved and the daytime frequency of urination assessed using the urinary log was significantly decreased. The consumption of SPE improved urinary frequency-related quality of life such as bother of urinary symptoms in healthy Japanese adults (UMIN000045334).

## Graphical abstract

**Graphical abstract:**
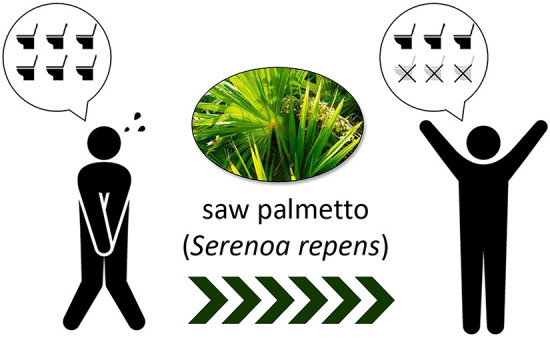
This is a visual representation of the abstract.

## Introduction

According to the Comprehensive Survey of Living Conditions in Japan (Ministry of Health Labour and Welfare, 2019), the ratio of Japanese individuals with subjective symptoms related to frequent urination (relative to 1000 population) was 33.0. By age group, the rate per 1000 population for those aged ≥65 years was 83.2, particularly 108.3 in men, which is the second most frequently reported subjective symptom after back pain. Furthermore, several reports have described that lifestyle diseases such as hyperglycemia, high triglyceride levels, and hypertension may be related benign prostatic hyperplasia (BPH) and lower urinary tract symptoms (LUTS) (Papaefstathiou et al., 2019; Qasrawi et al., 2022; Xiong et al., 2021). Lifestyle diseases are accompanied by inflammation, and which is considered an important factor in LUTS.

Recently, saw palmetto (*Serenoa repens*) has been attracting attention in Japan as a solution to urinary problems. Saw palmetto is an evergreen tree distributed from the Atlantic Coastal Plain to the Gulf Coast Stationary, and its berries, seeds, and shoots have been consumed by people (National Institutes of Biomedical Innovation, 2021). Fruit extracts are composed of 90% saturated and unsaturated fatty acids, as well as higher alcohols and sterols (Yamada and Ito, 2021). Because of its pharmacological effects such as 5α-reductase inhibition (Habib et al., 2005; Iehlé et al., 1995), antiandrogenic action (Sultan et al., 1984), and α1 receptor and muscarinic receptor binding activity (Goepel et al., 1999; Suzuki et al., 2007), it has been used as a drug or supplement to improve dysuria caused by BPH (European Medicines Agency, 2015).

In a prostate-free female rat model of urinary frequency, oral administration of a saw palmetto extract (SPE) resulted in prolonged voiding interval and increased single voiding volume and significant inhibition of transmural electrical stimulation and acetylcholine-induced contraction of bladder smooth muscle. Moreover, SPE antagonized the muscarinic receptors in the bladder, which ameliorated overactive voiding muscles in the female rat model. This action may be partially responsible for the blockade of muscarinic receptors (Yamada et al., 2020). Furthermore, SPE bound to the vanilloid receptor of bladder afferent sensory nerves (transient receptor potential vanilloid subtype 1; TRPV1) and inhibited its function (Yamada and Kato, 2022). Since bladder afferent sensory nerves transmit the sensation of the need to urinate to the brain (Birder et al., 2002), SPE may improve urinary frequency symptoms via blockade of TRPV1 in the bladder (Yamada and Kato, 2022). Additionally, animal studies have demonstrated that SPE exerts anti-inflammatory effects (Barakat et al., 2020a, 2020b). Those results *in vitro* and *in vivo* indicated SPE may be effective in both men and women with dysuria.

Although many elderly women suffer from dysuria (Durigon Keller et al., 2020; Lukacz et al., 2017), few clinical studies have examined the effect of SPE on dysuria symptoms in women. In a previous study, the efficacy and safety of a dietary supplement containing SPE as a main ingredient was examined in Japanese women with urinary problems (Yamada et al., 2022). SPE was administered for 12 weeks to 76 healthy Japanese women aged ≥50 years not diagnosed with overactive bladder (OAB) who had experienced frequent urination, nocturia, or urinary urgency for at least 2 months and had some degree of difficulty with urination. This previous study showed SPE improved urination frequency during the day (Yamada et al., 2022), possibly due to the blockade of muscarinic receptors and the inhibition of bladder afferent sensory nerves by SPE binding to vanilloid receptors.

Above these previous studies, SPE may improve dysuria with or without BPH. The purpose of this study was to examine the effect of SPE on the improvement of urinary frequency problems that present stress due to urinary urgency in daily life, mainly among healthy middle-aged and older men who are not diagnosed with BPH or OAB.

## Methods

### Participants

The inclusion criteria were shown as below:

- healthy Japanese men or women aged ≥50 years;
- concerned about frequency of urination;
- eligible to participate in the study by the physician;
- highly sensitive prostate specific antigen (PSA) less than 4 ng/mL at screening (examination before consumption; Scr);
- a score of urinary urgency of the OAB Symptom Score (OABSS) was <2, or total score of OABSS was <3 at Scr;
- an average daytime urinary frequency was ≥8 and <10 times in seven days prior to Scr; and
- “Frequent urination during the daytime hours” among “symptom bother” of the OAB questionnaire (OAB-q) Japanese version were relatively high in this study participants.

In order to exclude those who might affect the results of this study, exclusion criteria were established, including those with suspected chronic diseases or urinary-related disorders, regular users of pharmaceuticals, and those with food allergies (refer to the University Hospital Medical Information Network Clinical Trial Registry, UMIN000045334).

The participants were recruited via a website (https://www.go106.jp/) operated by ORTHOMEDICO Inc. (Japan). The study protocols were comprehensively explained to all participants at the office of ORHOMEDICO Inc. Furthermore, all participants provided informed consent before their participation in the study. No participants were part of the sponsor or funding companies. Although the study was conducted at Medical Corporation Seishinkai, Takara Clinic (Japan) that assessed the obtained data and took control of participants’ healthcare, examinations were conducted at both Medical Corporation Seishinkai, Takara Clinic and Nerima Medical Association, Minami-machi Clinic (Japan) as cooperating medical institutions.

### Intervention

Participants were administered treatments (SPE or placebo product) for 12 weeks: 320 mg of SPE (BGG Japan Co., Ltd, Japan) embedded in gelatin capsules (Yawata Saw Palmetto^®^, YAWATA CORPORATION, Japan) in the SPE group, while 320 mg of a glycerin fatty acid ester (food additive: Sunsoft No.707 (Taiyo Kagaku Co., Ltd, Japan) caprylic acid mono/diglyceride) embedded in gelatin capsules in the placebo group. SPE was prepared by supercritical extraction of dried saw palmetto fruit. These capsules were manufactured by an original equipment manufacturer. One capsule of SPE or placebo product was administered with water between after dinner and sleep once daily. The ethics committee declared that both capsules were identical in color, odor, and flavor.

### Outcomes

The schedule of this study is shown in Table 1. Efficacy assessments were conducted at Scr and examination 12 weeks after consumption (12wks) except for the daily record-forming. The daily record-forming efficacy assessments were recorded every day for 13 weeks between 1 week before Scr and 1 day before 12wks. If a participant encountered an adverse reaction or adverse event and took or used a pharmaceutical or over-the-counter drug, the participant was asked to report this information (e.g. types of adverse events and side effects, reason for use, type of drug, date, and dose) to the contract research organization (CRO).

**Table 1.**
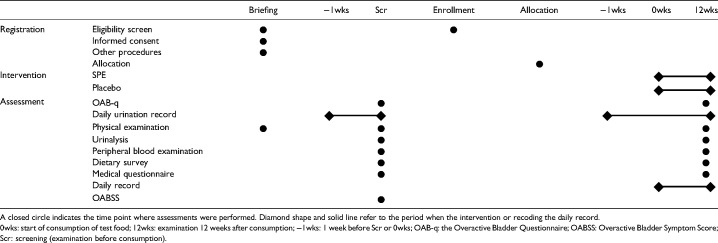
Schedule of enrollment, intervention, and assessment.

#### Primary outcome

The primary outcome was the measured value of “Frequent urination during the daytime hours” that was one of the items of “symptom bother” subscale of OAB-q Japanese version (Homma and Gotoh, 2006; Homma et al., 2006) at 12wks. OAB-q was a questionnaire for OAB specific-QOL (Coyne et al., 2002, 2005; Matza et al., 2005). Eight items constituting “symptom bother” were assessed on six-point scale from “1 = Not at all” to “6 = A very great deal” against “During the past 4 weeks, how bothered were you by …” and the higher the score, the more bothering the symptom.

#### Secondary outcomes

##### OAB-q Japanese version

The measured values of OAB-q Japanese version subscales, “symptom bother,” “Health-Related Quality of Life (HRQL) total score,” “Coping,” “Concern/worry,” “Sleep,” and “Social interaction” were assessed at 12wks and the change from Scr. This questionnaire comprised eight items measuring “symptom bother” and 25 items measuring “HRQL total score.” All items were assessed on a six-point scale. “HRQL total score” was the total score of “Coping,” “Concern/worry,” “Sleep,” and “Social interaction,” calculated from 0 to 100 and the higher the score was, the better the QOL.

##### Daily urination record

The daily frequency of urination was assessed by the daily urination record. The participants were required to record their daily urination every day for 13 weeks between 1 week before Scr and 1 day before 12wks. In this record, the average frequency of urination during daytime, at nighttime, and during the day were shown as the mean rounded to the second decimal place of each following period definitions: Period I was the duration between 1 week and 1 day before Scr. Period II (baseline) was the duration between 1 week and 1 day before the start of consumption. Periods III to XIII were every 7 days from the start of consumption. Period XIV was the last week (from day 78 to prior to 12wks). However, because the schedule of 12wks was sometimes shortened or extended due to the participant's convenience, period XIV was defined as 7 days prior to 12wks in this case.

The frequency of urination during the day was calculated by adding the frequency of daytime and nighttime urination.

Assessment points were the measured values in each time point from period III to XIV and changes from period II.

##### Safety evaluations

Safety evaluations were assessed in physical examination, urinalysis, and blood analysis. The collected samples in urinalysis and blood analysis were analyzed by LSI Medience Corporation (Japan). Evaluation items of physical examination, urinalysis, and blood analysis were same as Ishii et al. (2020).

The number of side effects and adverse events were counted. If the symptoms recognized as adverse event were expressed in the participants, the physician immediately took necessary and appropriate treatment and decided whether or not they could continue this study and the emergency key could be opened. In addition, the physician evaluated and reported in writing the relationship between the adverse event and the test food.

All participants were asked to complete a medical questionnaire and Calorie and Nutrition Diary (Suzuki et al., 2019) to allow for understanding of their health conditions at each examination. In addition, the participants were instructed to keep a daily record of consumption of the test food, health conditions, use of medications, and lifestyle.

### Sample size

To the best of our knowledge, no study has yet evaluated the effects of a 12-week intervention using SPE on the OAB-q in healthy Japanese adults. Hence, we assumed a large difference between the SPE and the placebo groups in the OAB-q, and an effect size (d) of 0.80 was used (Cohen, 1992). The sample size was calculated with an effect size (d) of 0.80, significance level (α) of 0.05, and statistical power (1 − β) of 0.80, respectively, thus requiring 26 participants per group. In order to maximize the statistical power (1 − β) as much as possible in the budget, the sample size was 31 participants per group, and statistical power (1 − β) was recalculated to be 87.3. Eventually, considering dropout or participants deviated protocol during this trial, the number of participants was 34 per group.

### Selection, randomization, and blinding

This study was a randomized, double-blind, placebo-controlled study, and allocation ratio was 1:1. Of the 139 participants who provided informed consent, 68 eligible participants were selected by the physician. The test food were provided to the CRO by the sponsor. After entering and verifying the data at Scr, an individual in charge of shipping, who is a member of the CRO, gave the code of the test products to an allocation controller who was not directly involved in the studies. Allocation was performed according to a computer-generated simple randomization list by this allocation controller. The allocation adjustment factors were sex, age, the score of “symptom bother” of the OAB-q at Scr, and the average of daytime urination frequency in period I. Participants were assigned to either the SPE group or the placebo group (n = 34 per group). The allocation table with the coded test products was provided only to the person in charge of shipping, who sent the test food to each participant according to the table. After the test food were shipped, the allocation table was kept in a secure location until the participants for analysis and statistical analysis methods were fixed. The group assignments were known only to the allocation controller. The allocation controller locked the allocation table until the key opening day.

### Statistical analysis

All statistical analyses in this study were two-sided, and the significance level was 5% with no adjustment for multiple comparisons. Data analyses were performed using Windows SPSS, version 23.0 (IBM Japan, Ltd, Japan). Quartiles were calculated using Microsoft Excel. The participants who markedly deviated protocol were excluded from analysis population. The participants’ age, height, weight, body mass index, body fat percentage, systolic and diastolic blood pressures, pulse rate, OABSS total score, and highly sensitive PSA for both groups were compared using Welch's *t-*test.

Postintervention efficacy assessments were performed the normality test and used an appropriate analysis. Measurements of the primary outcome and secondary outcomes except for the daily urination record were shown as minimum, maximum, interquartile range and mean ± standard deviation (SD). These were compared between groups using Mann–Whitney U test. Measurements of the daily urination record (mean ± SD) were compared between groups in only period II (as baseline) and period XIV. Welch's *t*-test was used for baseline, while a linear mixed model with baseline values as covariates and time points, groups, interaction between time points and groups, interaction between baseline values and time points, and participants as factors was used for the measured values and change from baseline in period XIV. In addition, group differences of estimated marginal means and 95% confidence intervals (CI) were shown. The above analysis methods were used in both per protocol set (PPS) and the male subgroup analysis set (MSS).

For the safety assessment items, the number of side effects and adverse events were counted and calculated their percentage per group and 95%CI of group difference. The percentage of cases for both groups were compared using chi-square test. In addition, the principal physician evaluated and checked the data case-by-case to confirm that there were no medical problems associated with ingestion of the test food.

## Results

### Study participants

**Figure 1.**
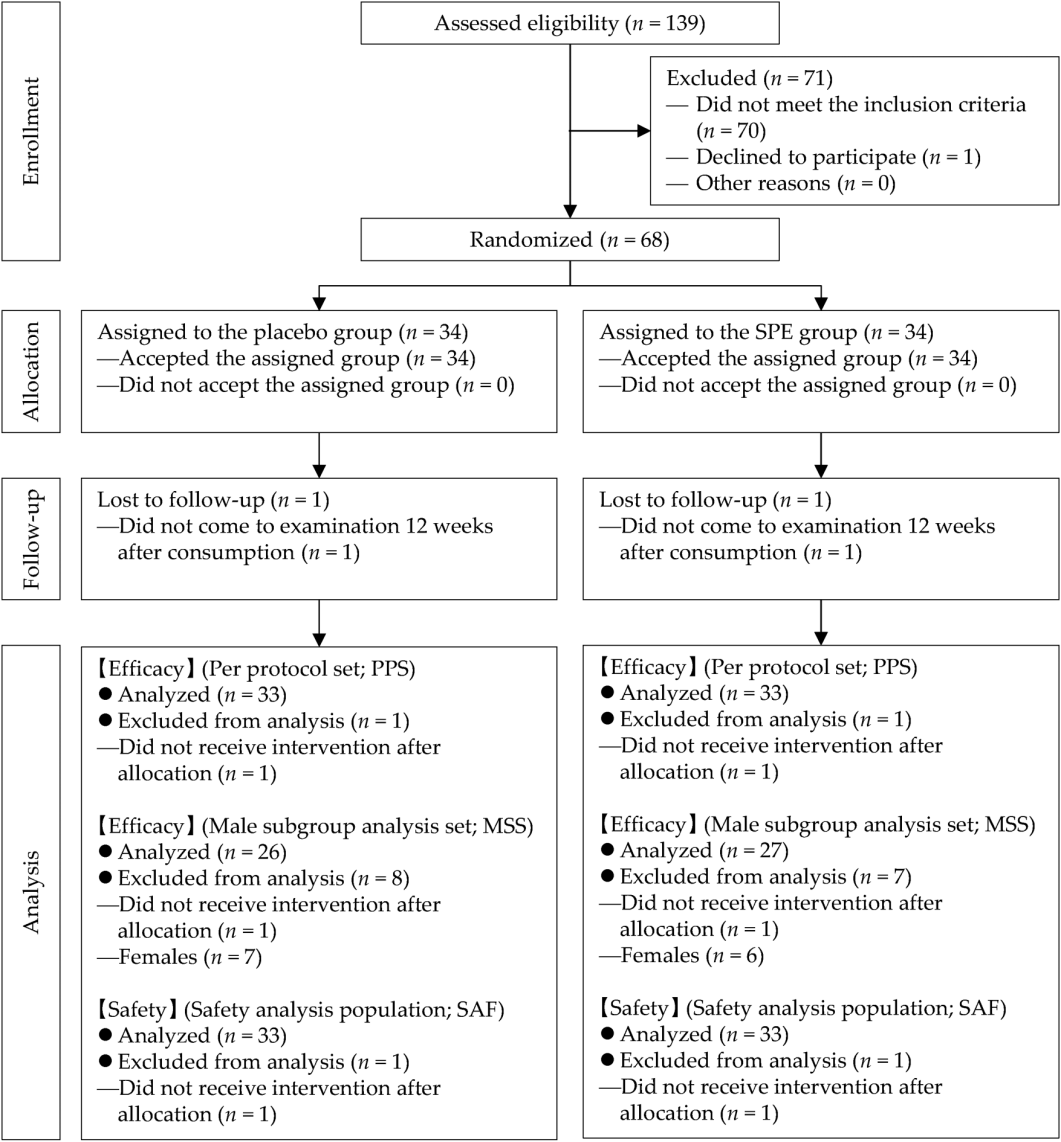
Flowchart of participants in this study. PPS: per protocol set; SPE: saw palmetto extract.

Figure 1 shows the flowchart of this study. We recruited participants from 2 September 2021, to 14 November 2021. The test period was from 2 September 2021, to 9 April 2022.

Two participants did not come to 12wks and excluded them from the analysis data set based on daily records and interviews. The final efficacy and safety analysis data sets were PPS and safety analysis population (SAF), respectively, both including 66 participants (33 in each group). The subgroup analysis set was MSS, and the number of participants was 53 (26 in the placebo group and 27 in the SPE group), excluding 13 female from the PPS.

The participants’ backgrounds are summarized in Table 2.

**Table 2.** Background characteristics of the participants.

	PPS/SAF				MSS				*p* value	
Items	Placebo group (*n* = 33)		SPE group (*n* = 33)		Placebo group (*n* = 26)		SPE group (*n* = 27)		PPS/SAF	MSS
	Mean	SD	Mean	SD	Mean	SD	Mean	SD		
Sex (male)	26 (78.8%)		27 (81.8%)		26 (100.0%)		27 (100.0%)		1.00	NA
Sex (female)	7 (21.2%)		6 (18.2%)		0 (0.0%)		0 (0.0%)		1.00	NA
Age (years)	57.7	5.8	58.1	5.9	58.3	5.7	58.6	6.1	0.82	0.86
Hight (cm)	167.6	7.4	166.0	6.8	170.2	5.6	168.0	5.3	0.34	0.16
Weight (kg)	65.3	11.6	67.3	12.0	69.2	9.6	70.2	10.9	0.49	0.72
BMI (kg/m^2^)	23.1	3.4	24.3	3.1	23.9	3.4	24.8	3.0	0.15	0.32
Body fat percentage (%)	21.1	5.2	24.6	5.4	20.5	5.3	23.5	5.0	0.011*	0.041*
Systolic blood pressure (mmHg)	120.3	16.4	129.7	18.2	123.7	15.8	133.4	17.0	0.031*	0.037*
Diastolic blood pressure (mmHg)	78.0	12.3	83.5	13.1	81.1	10.7	86.1	11.8	0.08	0.11
Pulse rate (bpm)	70.7	11.3	72.7	9.5	71.2	12.2	73.8	9.6	0.44	0.40
OABSS total score	2.8	1.3	3.2	1.5	2.7	1.3	3.1	1.4	0.23	0.26
High-sensitivity PSA (ng/mL)	0.9	0.7	0.9	0.7	1.1	0.6	1.1	0.6	0.81	0.96

Values of sex are shown as the number of cases and percentage of cases in each group. In other items, values are shown as mean and SD.

* *p* < 0.05.

BMI: body mass index; MMS: male subgroup analysis set; n: number of participants; NA: not available; OABSS: Overactive Bladder Symptom Score; PSA: prostate specific antigen; PPS: per protocol set; SAF: safety analysis population; SD: standard deviation.

### Subjective symptoms for urination

The results of the OAB-q are shown in Table 3.

**Table 3.** The results for the OAB-q in all participants (PPS analysis) and in the males of all participants (MSS) of SPE group and placebo group.

Data set	Items		Placebo group (*n* = 33)					SPE group (*n* = 33)					*p* value
			Min	Q1	Med	Q3	Max	Min	Q1	Med	Q3	Max	
PPS	Frequent urination during the daytime hours^†^	Baseline	2.0	2.0	2.0	3.0	5.0	2.0	2.0	3.0	3.0	5.0	0.96
		(mean ± SD)	(2.7 ± 0.8)					(2.6 ± 0.7)					
		12 wks	1.0	2.0	2.0	3.0	5.0	1.0	1.0	2.0	2.0	3.0	0.018*
		(Mean ± SD)	(2.3 ± 1.0)					(1.7 ± 0.6)					
	Symptom bother^‡^	Baseline	2.5	10.0	15.0	27.5	65.0	2.5	12.5	17.5	27.5	57.5	0.59
		(mean ± SD)	(21.7 ± 16.5)					(21.7 ± 13.6)					
		12 wks	0.0	7.5	12.5	25.0	55.0	0.0	7.5	10.0	22.5	52.5	0.34
		(Mean ± SD)	(19.0 ± 16.3)					(15.2 ± 12.3)					
	HRQL total score^‡^	Baseline	36.0	78.4	89.6	94.4	100.0	26.4	72.8	82.4	91.2	98.4	0.09
		(mean ± SD)	(84.0 ± 15.9)					(79.4 ± 15.9)					
		12 wks	38.4	84.8	92.0	96.0	100.0	52.0	83.2	92.0	94.4	99.2	0.56
		(mean ± SD)	(88.0 ± 12.4)					(86.7 ± 11.9)					
	Coping^‡^	Baseline	37.5	77.5	92.5	100.0	100.0	25.0	75.0	82.5	87.5	100.0	0.05
		(mean ± SD)	(84.5 ± 18.3)					(76.8 ± 20.2)					
		12 wks	35.0	82.5	90.0	100.0	100.0	52.5	77.5	90.0	95.0	100.0	0.45
		(mean ± SD)	(87.7 ± 13.9)					(86.0 ± 13.2)					
	Concern/worry^‡^	Baseline	31.4	85.7	94.3	97.1	100.0	28.6	80.0	91.4	97.1	100.0	0.38
		(mean ± SD)	(87.2 ± 17.4)					(85.9 ± 16.2)					
		12 wks	42.9	85.7	94.3	100.0	100.0	51.4	88.6	97.1	100.0	100.0	0.75
		(mean ± SD)	(89.8 ± 13.4)					(90.7 ± 13.3)					
	Sleep^‡^	Baseline	0.0	52.0	76.0	84.0	100.0	28.0	56.0	68.0	84.0	96.0	0.47
		(mean ± SD)	(69.6 ± 23)					(67.3 ± 19)					
		12 wks	36.0	68.0	84.0	92.0	100.0	36.0	72.0	80.0	84.0	96.0	0.32
		(mean ± SD)	(77.8 ± 19.2)					(76.1 ± 14.4)					
	Social interaction^‡^	Baseline	44.0	92.0	100.0	100.0	100.0	24.0	80.0	92.0	100.0	100.0	0.021*
		(mean ± SD)	(93.1 ± 12.8)					(86.4 ± 16.7)					
		12 wks	40.0	96.0	100.0	100.0	100.0	44.0	92.0	100.0	100.0	100.0	0.15
		(mean ± SD)	(96.4 ± 10.7)					(92.8 ± 12.5)					
MSS	Frequent urination during the daytime hours^†^	Baseline	2.0	2.0	2.0	3.0	4.0	2.0	2.0	3.0	3.0	5.0	0.57
		(mean ± SD)	(2.6 ± 0.8)					(2.7 ± 0.7)					
		12 wks	1.0	1.25	2.0	3.0	5.0	1.0	1.0	2.0	2.0	3.0	0.046*
		(mean ± SD)	(2.3 ± 1.1)					(1.7 ± 0.6)					
	Symptom bother^‡^	Baseline	2.5	10.0	15.0	25.0	45.0	2.5	12.5	20.0	27.5	57.5	0.32
		(mean ± SD)	(18.6 ± 13)					(22.3 ± 14.7)					
		12 wks	0.0	8.1	12.5	24.4	55.0	0.0	6.3	10.0	23.8	52.5	0.32
		(mean ± SD)	(19.1 ± 16.5)					(15.4 ± 13.3)					
	HRQL total score^‡^	Baseline	36.0	85.0	92.0	95.0	100.0	51.2	77.6	84.0	89.6	98.4	0.05
		(mean ± SD)	(86.2 ± 15.7)					(82.2 ± 11.7)					
		12 wks	38.4	85.0	92.4	98.4	100.0	58.4	86.4	92.8	94.8	97.6	0.57
		(mean ± SD)	(88.4 ± 13.2)					(88.0 ± 10.7)					
	Coping^‡^	Baseline	37.5	82.5	92.5	100.0	100.0	40.0	77.5	85.0	92.5	100.0	0.06
		(mean ± SD)	(87.5 ± 16.5)					(80.7 ± 17)					
		12 wks	35.0	83.1	90.0	100.0	100.0	62.5	86.3	92.5	96.3	100.0	0.61
		(mean ± SD)	(88.4 ± 14.8)					(88.5 ± 11)					
	Concern/worry^‡^	Baseline	31.4	89.3	97.1	99.3	100.0	60.0	82.9	91.4	97.1	100.0	0.17
		(mean ± SD)	(90.2 ± 16.1)					(89.0 ± 10.3)					
		12 wks	42.9	86.4	94.3	100.0	100.0	51.4	91.5	97.1	100.0	100.0	0.72
		(mean ± SD)	(90.1 ± 13.8)					(91.7 ± 12.6)					
	Sleep^‡^	Baseline	0.0	62.0	80.0	87.0	100.0	32.0	58.0	72.0	84.0	92.0	0.33
		(mean ± SD)	(70.9 ± 24.7)					(68.4 ± 17.8)					
		12 wks	36.0	69.0	84.0	92.0	100.0	36.0	70.0	80.0	86.0	92.0	0.25
		(mean ± SD)	(78.3 ± 19.8)					(75.7 ± 14.8)					
	Social interaction^‡^	Baseline	44.0	96.0	100.0	100.0	100.0	60.0	82.0	92.0	100.0	100.0	0.013*
		(mean ± SD)	(94.0 ± 13.7)					(88.9 ± 12.3)					
		12 wks	40.0	100.0	100.0	100.0	100.0	72.0	92.0	100.0	100.0	100.0	0.26
		(mean ± SD)	(96.2 ± 11.9)					(94.2 ± 9.7)					

Values are shown as minimum (Min), first quartile (Q1), median (Med), third quartile (Q3), maximum (Max), and mean ±  (SD).

† Six-point scale: “1 = Not at all”; “2 = A little bit”; “3 = Somewhat”; “4 = Quite a bit”; “5 = A great deal”; “6 = A very great deal”.

‡ Score from 0 to 100

* *p* < 0.05.

HRQL: Health-related quality of life; MMS: male subgroup analysis set; n: number of participants; OAB-q: the Overactive Bladder Questionnaire; PPS: per protocol set; SAF: safety analysis population; SD: standard deviation.

In PPS, the SPE group showed significantly lower values than the placebo group after the 12-week intervention (*p* = 0.018). PPS also showed that “Social interaction” in the SPE group was significantly lower than that in the placebo group at Scr but there was no significant group difference at 12wks. In any of the other subscale, there was no significant group difference at both Scr and 12wks.

In the subgroup analysis, MSS, the SPE group showed significantly lower values than the placebo group after the 12-week intervention (*p* = 0.046). As with PPS, in MSS, “Social interaction” in the SPE group was significantly lower only than that in the placebo group at Scr. After the intervention, there was no significant group difference. In any of the other subscale, there was no significant group difference at both Scr and 12wks.

### Daily urination record

**Figure 2.**
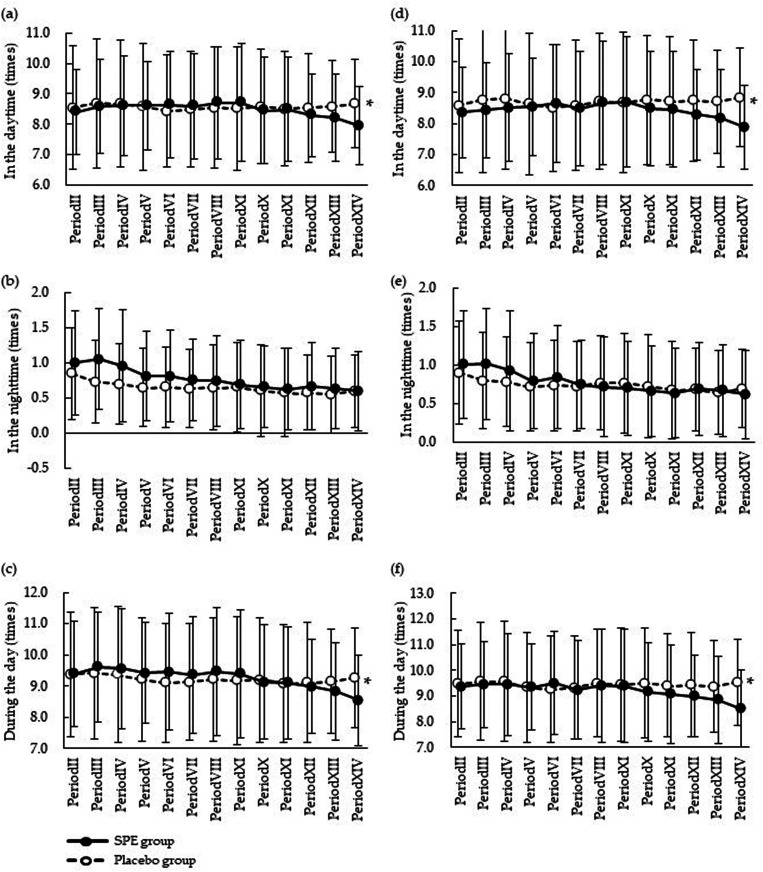
Changes in the average frequency of urination in all participants (PPS analysis). Period II (baseline) is defined as the duration between 1 week and 1 day before the start of consumption. Periods III to XIII are defined as every 7 days from the start of consumption. Period XIV is defined as 7 days prior to examination 12 weeks after consumption. Values are shown as mean and standard deviation. The average frequency of urination (a) during daytime, (b) at night time, and (c) during the day in PPS (*n* = 33 per group, respectively), and the average frequency of urination (d) during daytime, (e) at night time, and (f) during the day in MSS (SPE group, *n* = 27; placebo group, *n* = 26). * *p* < 0.05 vs. placebo group. MSS: male subgroup analysis set; PPS: per protocol set; SPE: saw palmetto extract.

The results of the daily urination record at baseline and period XIV are shown in Table 4, and the changes during intervention period are shown in Figure 2.

**Table 4.** The results for daily urination record in all participants (PPS analysis) and in the males of all participants (MSS) of the SPE group and placebo group.

Data set	The average frequency of urination		Placebo group (*n* = 33)		SPE group (*n* = 33)		Group differences	95%CI	*p* value
			Mean	SD	Mean	SD			
PPS	During daytime (times)	Baseline	8.5	2.0	8.4	1.4	−0.1	(−1.0, 0.7)	0.75
		Period XIV	8.7	1.5	7.9	1.3	−0.7	(−1.3, −0.1)	0.032*
		Change	0.1	1.7	−0.5	1.0	−0.7	(−1.3, −0.1)	0.032*
	At night time (times)	Baseline	0.8	0.7	1.0	0.7	0.2	(−0.2, 0.5)	0.37
		Period XIV	0.6	0.5	0.6	0.6	−0.1	(−0.3, 0.1)	0.42
		Change	−0.3	0.5	−0.4	0.5	−0.1	(−0.3, 0.1)	0.42
	During the day (times)	Baseline	9.4	2.0	9.4	1.7	0.0	(−0.9, 0.9)	0.96
		Period XIV	9.3	1.6	8.5	1.5	−0.7	(−1.4, −0.1)	0.031*
		Change	−0.1	1.8	−0.9	1.2	−0.7	(−1.4, −0.1)	0.031*
MSS	During daytime (times)	Baseline	8.6	2.2	8.4	1.4	−0.2	(−1.2, 0.8)	0.66
		Period XIV	8.8	1.6	7.9	1.4	−0.8	(−1.6, −0.1)	0.022*
		Change	0.3	1.8	−0.5	1.1	−0.8	(−1.6, −0.1)	0.022*
	At night time (times)	Baseline	0.9	0.7	1.0	0.7	0.1	(−0.3, 0.5)	0.59
		Period XIV	0.7	0.5	0.6	0.6	−0.1	(−0.3, 0.1)	0.18
		Change	−0.2	0.4	−0.4	0.5	−0.1	(−0.3, 0.1)	0.18
	During the day (times)	Baseline	9.5	2.1	9.4	1.7	−0.1	(−1.2, 0.9)	0.82
		Period XIV	9.5	1.7	8.5	1.5	−1.0	(−1.8, −0.2)	0.015*
		Change	0.1	1.8	−0.9	1.2	−1.0	(−1.8, −0.2)	0.015*

Values are shown as mean ± SD.

* *p* < 0.05.

MMS: male subgroup analysis set; n: number of participants; period XIV: 7 days prior to examination 12 weeks after consumption; PPS: per protocol set; SAF: safety analysis population; SD: standard deviation.

After the 12-week intervention in PPS, the measured values in period XIV of the SPE group were significantly lower than those of the placebo group (*p* = 0.032). Similarly, in period XIV, the change from baseline of the SPE group was significantly lower than that in the placebo group (*p* = 0.032). The measured values in the SPE group were significantly lower than those in the placebo group in period XIV (*p* = 0.031). Similarly, the change from baseline of the SPE group was significantly lower than that of the placebo group in period XIV (*p* = 0.031). There was no significant difference in the average frequency of urination at nighttime at any of the time points.

In MSS, the measured values in the SPE group were significantly lower than those in the placebo group in period XIV (*p* = 0.022). Similarly, the change from baseline of the SPE group was significantly lower than that in the placebo group in period XIV (*p* = 0.022). The measured values of the SPE group were significantly lower than those of the placebo group in period XIV (*p* = 0.015). Similarly, the change from baseline of the SPE group was significantly lower than that in the placebo group in period XIV (*p* = 0.015). There was no significant difference in the average frequency of urination in the nighttime at any of the time points.

### Safety evaluation

None of the participants experienced side effects during this trial. In contrast, adverse events were observed in six participants: four in the placebo group (allergic rhinitis, sneezing, gastritis, eye strain, sore throat, back pain, allergic conjunctivitis, eczema, headache, and anemia) and two in the SPE group (allergic rhinitis, swelling of fingers, and common cold). However, these participants were judged by the principal physician not to be attributable to the test food. Therefore, continuous consumption of the test food was deemed safe.

## Discussion

The measured value of “frequent urination during the daytime hours,” one of the questions comprising the “symptom bother” on the OAB-q at the 12-week postintake test, was significantly lower in the SPE group than that in the placebo group. This was the response to the question, “During the past 4 weeks, how bothered were you by frequent urination during the daytime hours?”, and the lower value means the smaller the degree of symptoms that bother (Homma and Gotoh, 2006; Homma et al., 2006). Additionally, the percentage of participants whose answers to that question were at least one level smaller after the intervention was 39.4% in the placebo group and 72.7% in the SPE group (group difference, 33.3%; 95% CI, 9.4% to 57.3%). For the average frequency of daily urination and diurnal urination, the measured value at period XIV (the last week of the intervention period) and the change value from period I or period II to period XIV were significantly lower in the SPE group than those in the placebo group (data not shown). These results indicated that SPE clinically improve the degree of distress caused by frequent urination during the day via decreasing urination frequency.

SPE binds to TRPV1 in the afferent sensory nerves of the bladder (Yamada and Kato, 2022). In female LUTS patients with urinary urgency, TRPV1 mRNA expression in the bladder epithelium in the bladder triangle was inversely correlated with bladder capacity (Liu et al., 2007). Moreover, taking SPE 320 mg/day for 12 weeks improved subjective symptoms related to urination in over 50 years Japanese women experiencing frequent urination, nocturia, and urinary urgency for at least 2 months (Yamada et al., 2022). Consistent with these reports, our study confirmed a trend toward improvement with SPE in the analysis of the female subgroup (data not shown). Since TRPV1 is expressed in the bladder epithelium of both women and men (Nilius et al., 2007), we concluded that SPE reduced urinary urgency and frequency of urination through binding to TRPV1.

The bladder and prostate play an important role in the control of urination and urinary storage, and SPE act on these organs. Bladder smooth muscle controls bladder function; this muscle relaxes during collecting and storing and then contracts during micturition (Morita, 1997; Ukai et al., 2003; Yoshimura, 2003). The smooth muscle of the bladder contracts because the acetylcholine released from the parasympathetic nervous system binds to the muscarinic receptors (Yamaguchi, 2003; Yoshimura, 2003). SPE binds the muscarinic receptors and act like antagonists (Oki et al., 2005), which reveals that the main components of SPE (lauric acid, oleic acid, myristic acid, and linoleic acid) bind to muscarinic receptors (Abe et al., 2009a, 2009b). In the prostate gland, urinary drainage and storage are impaired by functional obstruction (functional narrowing of the urethra due to contraction of the prostate smooth muscle) and/or mechanical obstruction (physical obstruction of urine drainage due to enlargement of the prostate gland) (Kawabe, 2006). The prostatic smooth muscle contracts due to hypertonia caused by sympathetic α1 receptor stimulation (Schwinn and Roehrborn, 2008), while prostate enlargement is caused by dihydrotestosterone, which is the male hormone testosterone converted by 5α-reductase (Morita, 1997). The α1 receptor binding ability of SPE (Oki et al., 2005) has been attributed to the binding of the lauric acid, oleic acid, myristic acid, and linoleic acid to α1 receptors (Abe et al., 2009a, 2009b). SPE also suppresses the production of dihydrotestosterone by inhibiting 5α-reductase activity (Habib et al., 2005). An *in vivo* study showed that oral ingestion of SPE results in an increased bladder capacity and prolonged urinary intervals (Oki et al., 2005; Suzuki et al., 2007). Therefore, SPE can reduce the hypercontraction of the smooth muscle in the bladder and prostate and ameliorate the mechanical obstruction due to prostate enlargement.

Given these evidences, we thought that (1) SPE improved urination and storage symptoms by inhibiting bladder tone, reducing the frequency of urination, and alleviating the degree to which people felt troubled by the frequency of urination during the day in women and (2) SPE improved urinary and storage symptoms in men by suppressing bladder and prostate tension since the prostate is a male-specific organ. The improvement in subjective symptoms observed in this study is in agreement with previous studies (Ishii et al., 2020; Yamada et al., 2022), indicating that taking SPE is useful in improving QOL related to urination in both men and women.

While the PPS analysis can be useful in demonstrating the effects of the intervention except those of protocol deviations and noncompliance, its results cannot maintain the comparability between groups established by randomization and the final results may reflect baseline group differences (Sedgwick, 2015). Although the analytical dataset used to evaluate efficacy was the PPS in this study, there was no significant difference between groups at baseline regarding “urinating multiple times during the day” of the OAB-q, and medically meaningful group differences were observed at 12 wks. Thus, we consider that the PPS analysis data also prove the effect of the intervention.

In conclusion, the continuous intake of SPE 320 mg/day for 12 weeks in healthy Japanese men and women aged ≥50 years who had problems with urination frequency provide a significant improvement in frequent urination during the daytime hours among the symptom bother and a significant reduction in the daily frequency of urination, particularly during the daytime.
